# Mild Obesity, Physical Activity, Calorie Intake, and the Risks of Cervical Intraepithelial Neoplasia and Cervical Cancer

**DOI:** 10.1371/journal.pone.0066555

**Published:** 2013-06-12

**Authors:** Jae Kwan Lee, Kyeong A. So, Chandrika J. Piyathilake, Mi Kyung Kim

**Affiliations:** 1 Department of Obstetrics and Gynecology, Korea University College of Medicine, Seoul, Korea; 2 Department of Nutrition Sciences, University of Alabama at Birmingham, Birmingham, Alabama, United States of America; 3 Cancer Epidemiology Branch, National Cancer Center, Goyang, Korea; Baylor College of Medicine, United States of America

## Abstract

**Objective:**

We investigated whether obesity, physical activity, and calorie intake are associated with the risks of cervical intraepithelial neoplasia (CIN) and cervical cancer.

**Methods:**

We enrolled 1125 women (age, 18–65 years) into a human papillomavirus cohort study established from 2006 to 2012. Multinomial logistic regression models were used to estimate crude and multivariate odds ratios (ORs) and the corresponding 95% confidence intervals (95% CIs), and to assess whether body mass index (BMI), height, weight, total calorie intake, and physical activity were associated with the risks of CIN and cervical cancer.

**Results:**

Cervical cancer risk was positively associated with BMI and inversely associated with physical activity. When compared with women with a normal BMI (18.5–23 kg/m^2^), the multivariate ORs (95% CIs) for those overweight (23–25 kg/m^2^) and mild obesity (≥25 kg/m^2^) were 1.25 (0.79–2.00) and 1.70 (1.10–2.63), respectively. When compared with women with the lowest tertile of physical activity (<38.5 MET-hours/week), the ORs (95% CIs) for cervical cancer were 0.95 (0.61–1.48) and 0.61 (0.38–0.98) for women with medium physical activity (38.5–71.9 MET-hours/week) and those with high physical activity (72 MET-hours/week), respectively (p for linear trend  = 0.03). The CIN2/3 risk was inversely associated with physical activity after adjustment for confounders. Compared with women with low physical activity (< 38.5 MET-hours/week), the ORs (95% CIs) for CIN2/3 were 0.64 (0.40–1.01) and 0.58 (0.36–0.93) for the medium and high physical activity groups, respectively (p for linear trend  = 0.02). Total calorie intake was not statistically associated with the risks of CIN and cervical cancer after adjustment for confounders.

**Conclusion:**

Our results indicate that in addition to screening for and treatment of CIN, recommendations on the maintenance of an appropriate BMI with an emphasis on physical activity could be an important preventive strategy against the development of cervical cancer.

## Introduction

Although human papillomavirus (HPV) infection is a major risk factor for cervical carcinogenesis, cervical cancer is a multifactorial disease [1], [2]. Cofactors known to contribute to the progression of HPV infection to neoplasia include oral contraceptive use, multiparity, smoking, host immune response, nutritional status, and concomitant inflammation due to infectious diseases such as *Chlamydia trachomatis* and herpes simplex virus [3]–. In addition, several factors are likely to play an important role in determining a person’s risk of developing cervical cancer. Specifically, obesity, body size, physical activity, calorie intake, smoking, alcohol, and occupational exposures may contribute to the progression of or protection against cervical carcinogenesis [6]. Obesity is known to be linked to health risks; however, the mechanism by which the body mass index (BMI) becomes a risk factor for all cancer and site-specific cancers is not yet fully elucidated. The plausible biological mechanisms for the increased risk include inflammation-associated carcinogenesis and increased levels of endogenous hormones (sex steroids, insulin, and insulin-like growth factor I) [7].

Previous epidemiologic studies have consistently shown associations between obesity and increased risks for cancers of the endometrium, kidney, gallbladder (in women), breast (in postmenopausal women), and colon (particularly in men) [7]. However, data on cervical cancers are scarce or inconsistent [8], [9]. Indeed, some studies have shown an association between obesity and the incidence of cervical cancer [10]–[12], whereas other studies did not find such an association [13]. This lack of consistency may be attributable to the limited number of studies (especially those with prospective cohorts), limited range and variable categorization of overweight and obesity among studies (especially, Asian women are relatively leaner than Western women), failure to consider all potential confounders, and possible real differences between the effects of obesity on the incidence and mortality rate of cervical cancer.

Some epidemiologic studies have reported beneficial effects of physical activity on the risk of cancer [14]–[20]. The second report of the World Cancer Research Fund/American Institute for Cancer Research concluded that all forms of physical activity protect against cancer, including colon cancer, postmenopausal breast cancer, and endometrial cancer, in relation to or independently of weight gain or being overweight and obese [21]. Height, weight, and calorie intake are also associated with gynecologic cancer risk [19], [22]–[27]. Several studies have reported an association between physical activity, height, weight, and calorie intake and ovarian, endometrial, and breast cancers; however, epidemiologic evidence on the relation between physical activity, body size, calorie intake, and cervical intraepithelial neoplasia (CIN) and cervical cancer is limited [6], [15]–[17], [19], [20], [22]–[27].

Therefore, the objective of the present study was to determine whether obesity, physical activity, or calorie intake is associated with the risks of CIN and cervical cancer.

## Materials and Methods

### Subject Recruitment

This study was conducted among the women participating in a prospective HPV cohort study at the National Cancer Center (NCC) in Korea from March 2006 up to the present, which has been described in detail previously [28]–[30]. Briefly, subjects were randomly selected from the gynecologic oncology clinics of 5 university hospitals in Korea. Women were eligible to participate if, at the time of enrollment, they were sexually active or seeking birth control, aged 18 to 65 years, not pregnant, had an intact uterus, had no current referral for hysterectomy, and had no history of treatment for CIN within the last 18 months. Exclusion criteria included a history of gynecological cancers such as ovarian or endometrial cancer; insufficient data on the questionnaire; inadequate blood for evaluation; a chronic disease such as liver cirrhosis, renal failure, or cardiovascular disease; drug dependency; or psychological problems. All study participants signed an informed consent form as required by the institutional review boards. The study was approved by the ethics committees of the NCC (NCCNCS-06-062) and of each study center. Upon study entry, participants were interviewed by a trained interviewer with a questionnaire about the risk factors for cervical cancer. Participants also underwent a physical and gynecological examination, as well as Hybrid Capture 2 (HC2) and Papanicolaou (Pap) smear tests. The HC2 assay was used to test patients for HPV DNA, and the viral load (relative light units/positive control [RLU/PC]) was also measured. Follow-up visits were scheduled every 4 months in the first year and every 6 months thereafter. At each visit, participants answered a questionnaire on lifestyle and dietary behavior, underwent a pelvic examination, and provided cervical specimens. Colposcopic examinations and histologic verifications were performed at baseline and during follow-up visits in all women.

The present analyses focused on the baseline data only. Of the 1353 women enrolled, we excluded 171 women with insufficient questionnaire data or missing baseline blood sample, and 57 women with a chronic disease such as liver cirrhosis or cardiovascular disease. After the exclusions, 1125 women who completed all measurements remained for the analyses. The histopathological diagnoses included 190 cases of CIN1, 192 cases of CIN2/3, and 200 cases of cervical cancer. Control subjects (n = 543) were those who had a normal Pap smear on the day of recruitment and without any history of abnormal Pap smears.

### Liquid-based Cytology

Exfoliated cells obtained from the uterine cervix were immediately rinsed in a vial containing PreservCyt solution (Cytyc Corporation, Marlborough, MA), and then the vial was placed in a Thin Prep processor (Cytyc Corporation). Cytological grading was based on the Bethesda classification system [19] for Pap smear reports. All women with repeated diagnosis of atypical squamous cells of undetermined significance (ASCUS) or with cytological evidence of atypical squamous cells, cannot exclude high-grade lesions (ASC-H), low-grade squamous intraepithelial lesion (LSIL), and high-grade squamous intraepithelial lesion (HSIL) were diagnosed by histopathological examination of specimens obtained from colposcopic-guided biopsies. Histological grading was according to the World Health Organization (WHO)-accepted criteria.

### Detection of HPV DNA

The HC2 system (Digene, Gaithersburg, MD) was used for HPV detection. This technology is a signal-amplified hybridization antibody capture assay that uses chemiluminescent detection with a specific HPV RNA probe cocktail for carcinogenic high-risk HPV types (16, 18, 31, 33, 35, 39, 45, 51, 52, 56, 58, 59, and 68). Cervical specimens for HPV DNA testing were collected with a cervical sampler, and the HPV test was performed according to the manufacturer’s instructions. The RLUs measured on a luminometer denoted the presence or absence of HPV DNA sequences in the specimen. The sample was classified as positive when the RLU/PC ratio (RLU of the specimen/mean RLU of 3 PCs) was ≥1 pg/mL. When the RLU was less than the cutoff value, the sample was considered to be negative for that specific HPV DNA sequence or to have HPV DNA levels below the detection limit of the assay.

### Data Collection

Using a structured questionnaire designed for this study, a wide range of lifestyle and sociodemographic characteristics of the patients were collected upon enrollment, including cigarette smoking, alcohol consumption, exercise habits, education, occupation, medical history, reproductive and menstrual history, exogenous hormone use with a detailed time frame of exposure, and family history of cervical cancer and other cancers. Pathological and clinical data were also collected. Self-reported body height (cm) and weight (kg) were collected at the time of diagnosis. The BMI, computed as the weight in kilograms divided by the square of the height in meters, was categorized using the WHO [31] definition of obesity for Asians (underweight, <18.5 kg/m^2^; normal weight, 18.5–23 kg/m^2^; overweight, 23–25 kg/m^2^; obesity, ≥25 kg/m^2^).

A structured physical activity questionnaire that estimates an individual’s time spent on leisure-time physical activity was used to document habitual physical activity. Subjects specified how many hours of a 24-hour day they spent doing vigorous, moderate, and light activities over the past 7 days [32], [33]. Physical activity was divided into 3 categories of activity and metabolic equivalent of energy expenditure (MET) according to the WHO Global Strategy on Diet, Physical Activity, and Health: (1) light-intensity physical activities: require light physical effort and correspond to a consumption of 2.1–2.9 METs (e.g., driving a car, sewing, doing laboratory work, or reading a book); (2) moderate-intensity physical activities: physically tiring but do not cause breathlessness and require a consumption of 3.0–5.9 METs (e.g., washing dishes, moving light boxes, or light cycling); and (3) vigorous-intensity physical activities: cause sweating, increased heartbeat, and breathlessness, requiring a consumption of ≥6 METs (e.g., climbing stairs while carrying heavy bags, jogging, running, or doing aerobics). The subjects specified how many hours of a 24-hour day they spent doing vigorous, moderate, and light activities for a usual 1 week. Additionally, MET-hours per week was estimated for each woman by multiplying together the number of hours per week a woman spent in a particular activity and the estimated MET score for that activity based on the Compendium of Physical Activities [34]. To estimate the activity levels, the total physical activity was categorized by the assigned MET-hours/week value to each of the light-, moderate-, and vigorous-intensity activities: 2.5, 5, and 7.0, respectively.

Each participant’s usual dietary intake was recorded, with details of food intake during the year before enrollment, using a 95-item semiquantitative food-frequency questionnaire (SQFFQ) [35], including the usual frequency of consumption and typical portion sizes. The frequency of intake in the SQFFQ was classified into 9 categories: almost never, once per month, 2–3 times per month, 1–2 times per week, 3–4 times per week, 5–6 times per week, once per day, twice per day, and 3 times per day. The standard portion size of each dish item per meal was determined according to the Korean Ministry of Health and Welfare portion size booklet [36]. In the SQFFQ, portion size was divided into 3 categories: small (one-half the medium portion), medium, and large (≥1.5 times the medium portion). The medium intake was determined by the mean amount for the study subjects. The usual food intakes derived from the FFQ were calculated by multiplying the frequency of consumption with the daily portion size for each food group. The nutrient intake for each food item was calculated using the Diet Analysis program (version 4.0; ESHA Research, Salem, OR) for nutrients. Photographs showing the most representative serving sizes were used to assist subjects in estimating the serving sizes.

### Statistical Analysis

Distributions of general characteristics are presented as means ± standard deviations for continuous variables and as frequencies for categorical variables. Means were compared using Student’s t tests, while χ^2^ tests were used to compare frequencies. Multinomial logistic regression models were used to estimate crude and multivariate odds ratios (ORs) and the corresponding 95% confidence intervals (95% CIs) for CIN1, CIN2/3, and cervical cancer, and to assess whether BMI, height, weight, total calorie intake, and physical activity were associated with the risks of CINs and cervical cancer. The effects of potential confounding factors such as age (continuous variable), parity (0, 1, 2, or ≥3), smoking habit (never vs. ever), menopausal status (premenopause vs. postmenopause), alcohol consumption (never vs. ever), HPV infection (negative vs. positive), and oral contraceptive use (never vs. ever) were considered with multivariate adjustments. The model estimated 3 ORs simultaneously for each risk factor evaluated: the OR for CIN1 versus control, CIN2/3 versus control, and cervical cancer versus control [37], [38]. The test for linear trends was calculated using the median values for each risk factor as a continuous variable. Total calorie intake, height, and weight were categorized into tertiles for analyses based on the distribution of each value among control subjects, with the lowest tertile as the reference category. All analyses were conducted using Stata (Version 10.0; Stata Corporation, College Station, TX).

## Results

The sociodemographic, reproductive, anthropometric, and lifestyle characteristics of patients with CIN and cervical cancer, and those of controls are presented in Table 1. Women with cervical cancer were more likely to be older and those with CINs were likely to be younger than the controls (p<0.01). A higher prevalence of alcohol drinkers was observed among women with CIN1 and CIN2/3 when compared with controls and those with cervical cancer (p<0.01). Patients with cervical cancer had a greater number of childbirths compared with patients with CIN and controls (p<0.01). About the use of oral contraceptives and menopausal status, there were significant differences between patients with CIN/cervical cancer and controls (p<0.05). However, total calorie intake, cigarette smoking, and HPV infection status were not different between patients with CIN/cervical cancer and controls. Overall, patients with cervical cancer were shorter and heavier than controls (p<0.01). The mean BMI was higher in patients with cervical cancer than in controls and in patients with CIN (p<0.01).

**Table 1:** Sociodemographic, reproductive, anthropometric, and lifestyle characteristics of study subjects.

Variables	Normal (n = 543)	CIN1 (n = 190)	CIN2/3 (n = 192)	Cervical cancer (n = 200)	p^1^
Age (y), mean ± SD	45.1±10.3^b^	41.0±11.2^c^	41.0±10.1^c^	51.0±11.9^a^	<0.01
≤29, n (%)	36 (7)	31 (16)	26 (14)	5 (2)	
30–39	128 (23)	62 (33)	68 (35)	28 (14)	
40–49	195 (36)	53 (28)	64 (33)	71 (35)	
50–59	136 (25)	32 (17)	25 (13)	47 (24)	
≥60	48 (9)	12 (6)	9 (5)	49 (25)	<0.01
Cigarette smoking, n (%)					
Non-smoker	489 (90)	162 (85)	161 (84)	173 (87)	
Smoker	54 (10)	28 (15)	31 (16)	27 (13)	0.08
Alcohol consumption, n (%)					
Non-drinker	276 (51)	54 (28)	72 (37)	104 (52)	
Drinker	267 (49)	136 (72)	120 (63)	96 (48)	<0.01
Ever use of oral contraceptive, n (%)					
Never	465 (86)	157 (83)	147 (77)	164 (82)	
Current/former	78 (14)	33 (17)	45 (23)	36 (18)	0.03
Menopausal status, n (%)					
Premenopausal	344 (63)	148 (78)	153 (80)	71 (35)	
Postmenopausal	199 (37)	42 (22)	39 (20)	129 (65)	<0.01
HPV infection positive, n (%)	131 (47)	58 (57)	36 (52)	15 (47)	0.36
No. of childbirths, n (%)	1.89±1.15^b2^	1.69±1.20^b^	1.73±1.13^b^	2.53±1.39^a^	<0.01
0	82 (15)	47 (25)	37 (19)	12 (6)	
1	68 (13)	15 (8)	26 (14)	17 (9)	
2	275 (51)	91 (48)	94 (49)	92 (46)	
≥3	117 (21)	37 (19)	35 (18)	78 (39)	<0.01
Height (cm), mean ± SD	158±5.25^b^	159±4.93^a^	158±5.46^b^	156±5.22^c^	<0.01
Weight (kg), mean ± SD	56.1±7.53^bc^	56.6±8.14^ab^	54.9±7.22^c^	57.8±9.29^a^	<0.01
BMI (kg/m^2^), mean ± SD	22.5±2.90^b^	22.3±3.12^b^	22.0±3.15^b^	23.6±3.68^a^	<0.01
Total calorie intake (kcal/day), mean ± SD	1,854±596^a^	1,944±661^a^	1,938±588^a^	1845±625^a^	0.06
Leisure-time physical activities (MET-hours/week), mean ± SD	62.5±44.2^a^	58.1±46.0^ab^	52.2±35.9^b^	64.0±76.0^a^	0.05

CIN, cervical intraepithelial neoplasia; HPV, human papillomavirus; BMI, body mass index; MET, metabolic equivalent; SD, standard deviation.

1 Analysis of variance for continuous variables and χ^2^ test for categorical variables.

2 Values in rows with different superscripts are significantly different from each other.

The associations of height, weight, and BMI with the risk of CIN1, CIN2/3, and cervical cancer are shown in Table 2. BMI was associated with an increased risk of cervical cancer. When compared with women with a normal BMI (18.5–23 kg/m^2^), the multivariate-adjusted ORs for those overweight (23–25 kg/m^2^) and mild obese (≥25 kg/m^2^) were 1.25 (0.79–2.00) and 1.70 (1.10–2.63), respectively (p for linear trend  = 0.02). No significant association of height, weight, and BMI was found in patients with CIN1 and CIN2/3.

**Table 2:** Association of height, weight, and BMI with the risk of CIN1, CIN2/3, and cervical cancer.

	Control (n = 543) (%)	CIN1 (n = 190)			CIN2/3 (n = 192)			Cervical cancer (n = 200)		
		%	Crude OR (95% CI)	Multivariate OR^1^ (95% CI)	%	Crude OR (95% CI)	Multivariate OR^1^ (95% CI)	%	Crude OR (95% CI)	Multivariate OR^1^ (95% CI)
Height (cm)										
<157	36	24	1 (ref.)	1 (ref.)	36	1 (ref.)	1 (ref.)	48	1 (ref.)	1 (ref.)
157 to <160	35	38	1.69 (1.11–2.58)	1.60 (1.00–2.57)	33	0.95 (0.64–1.41)	0.78 (0.49–1.22)	32	0.72 (0.50–1.05)	0.93 (0.61–1.42)
≥160	29	38	2.01 (1.31–3.08)	1.44 (0.85–2.43)	31	1.09 (0.73–1.64)	0.76 (0.46–1.26)	20	0.52 (0.34–0.79)	0.85 (0.51–1.40)
p for trend			<0.01	0.15		0.69	0.26		<0.01	0.51
Weight (kg)										
<53	33	35	1 (ref.)	1 (ref.)	40	1 (ref.)	1 (ref.)	34	1 (ref.)	1 (ref.)
53 to <59	33	29	0.83 (0.55–1.25)	0.91 (0.55–1.57)	34	0.85 (0.58–1.25)	1.03 (0.65–1.64)	25	0.77 (0.50–1.16)	0.64 (0.40–1.02)
≥58	34	36	1.01 (0.68–1.50)	1.16 (0.73–1.85)	26	0.65 (0.43–0.97)	0.73 (0.45–1.18)	41	1.22 (0.83–1.79)	0.97 (0.63–1.48)
p for trend			0.96	0.51		0.03	0.2		0.28	0.96
BMI (kg/m^2^)										
18.5–22.9	56	51	1 (ref.)	1 (ref.)	56	1 (ref.)	1 (ref.)	43	1 (ref.)	1 (ref.)
<18.5	6	11	2.39 (1.30–4.40)	2.38 (1.04–5.48)	11	2.15 (1.17–3.94)	2.23 (1.00–4.98)	5	1.27 (0.59–2.72)	2.39 (0.97–5.91)
23–24.9	19	18	1.06 (0.68–1.66)	1.28 (0.76–2.14)	19	1.01 (0.65–1.56)	1.42 (0.87–2.31)	21	1.42 (0.93–2.19)	1.25 (0.79–2.00)
≥25	19	20	1.17 (0.75–1.80)	1.45 (0.88–2.42)	14	0.74 (0.46–1.20)	0.85 (0.49–1.47)	31	2.12 (1.43–3.15)	1.70 (1.10–2.63)
p for trend			0.52	0.12		0.34	0.86		<0.01	0.02

ORs, odds ratios; 95% CIs, 95% confidence intervals; CIN, cervical intraepithelial neoplasia; BMI, body mass index.

1 Adjusted for age (years, continuous), parity (0, 1, 2, and ≥3), smoking habit status (never vs. ever), menopause (premenopause vs. postmenopause), human papillomavirus infection status (negative vs. positive), alcohol consumption status (never vs. ever), and oral contraceptive use (never vs. ever).

The crude and multivariate ORs of CIN1, CIN2/3, and cervical cancer with respect to total calorie intake and physical activity are shown in Table 3. After adjustment for confounders, physical activity was inversely associated with CIN2/3. Compared with women with low physical activity (<38.5 MET-hours/week), the ORs (95% CIs) for CIN2/3 were 0.64 (0.40–1.01) and 0.58 (0.36–0.93) for those with medium physical activity (38.5–71.9 MET-hours/week) and high physical activity (≥72 MET-hours/week), respectively (p for linear trend  = 0.02). Physical activity also reduced the risk of cervical cancer. Compared with women with the lowest tertile of physical activity (<38.5 MET-hours/week), the ORs (95% CIs) for cervical cancer were 0.95 (0.61–1.48) and 0.61 (0.38–0.98) for those with medium physical activity (38.5–71.9 MET-hours/week) and high physical activity (≥72 MET-hours/week), respectively (p for linear trend  = 0.03).

**Table 3:** Association of total calorie intake and physical activity with the risk of CIN1, CIN2/3, and cervical cancer.

	Control (n = 543) (%)	CIN1 (n = 190)			CIN2/3 (n = 192)			Cervical cancer (n = 200)		
		%	Crude OR (95% CI)	Multivariate OR^1^ (95% CI)	%	Crude OR (95% CI)	Multivariate OR^1^ (95% CI)	%	Crude OR (95% CI)	Multivariate OR^1^ (95% CI)
Total calorie intake (kcal/day)										
<1571	34	27	1 (ref.)	1 (ref.)	22	1 (ref.)	1 (ref.)	32	1 (ref.)	1 (ref.)
1571 to <1986	33	33	1.19 (0.78–1.82)	1.04 (0.63–1.72)	43	1.95 (1.28–2.99)	1.98 (1.22–3.22)	35	1.11 (0.75–1.65)	1.27 (0.82–1.99)
≥1986	33	40	1.46 (0.97–2.20)	1.26 (0.77–2.14)	35	1.62 (1.05–2.51)	1.25 (0.74–2.10)	33	1.02 (0.68–1.52)	1.35 (0.85–2.15)
p for linear trend			0.06	0.34		0.04	0.52		0.94	0.19
Leisure-time physical activities (MET-hours/week)										
<38.5	30	37	1 (ref.)	1 (ref.)	43	1 (ref.)	1 (ref.)	33	1 (ref.)	1 (ref.)
38.5–72	36	35	0.77 (0.52–1.15)	0.98 (0.61–1.58)	32	0.60 (0.41–0.89)	0.64 (0.40–1.01)	39	0.95 (0.65–1.40)	0.95 (0.61–1.48)
≥72	34	28	0.68 (0.45–1.23)	0.82 (0.50–1.34)	25	0.51 (0.34–0.77)	0.58 (0.36–0.93)	28	0.73 (0.48–1.10)	0.61 (0.38–0.98)
p for linear trend			0.06	0.42		<0.01	0.02		0.13	0.03

ORs, odds ratios; 95% CIs, 95% confidence intervals; CIN, cervical intraepithelial neoplasia; MET, metabolic equivalent.

1 Adjusted for age (years, continuous), parity (0, 1, 2, and ≥3), smoking habit status (never vs. ever), menopause (premenopause vs. postmenopause), human papillomavirus infection status (negative vs. positive), alcohol consumption status (never vs. ever), and oral contraceptive use (never vs. ever).

## Discussion

Several studies have examined the association between obesity and cervical cancer, but the results are limited and inconclusive [10]–[13]. The expert working group of the International Agency for Research on Cancer concluded that “the evidence is too limited to allow any conclusion on the relationship between BMI and the risk of cervical cancer” [39]. The second report of the World Cancer Research Fund/American Institute for Cancer Research also suggested that data from qualified studies about the relation between body fatness and the risk of cervical cancer were too few to allow reaching valid conclusions [21]. However, some studies have reported that the mean BMI of patients with cervical carcinoma was significantly higher than that of healthy female controls, and suggested that obesity seems to increase the risk of cervical cancer [10], [11]. In our study, compared with women with a normal BMI (18.5–23 kg/m^2^), the ORs for cervical cancer were 1.25 for overweight (23–25 kg/m^2^) and 1.70 for mild obesity (≥25 kg/m^2^), suggesting that obesity may increase the cervical cancer risk. Several factors may explain the observed results. First, several studies have reported that obese women might receive screening for cervical cancer less frequently than normal-weight women, increasing the likelihood of obese women developing cervical cancer [7], [40]. A recent systemic review and meta-analysis, in fact, has documented that obesity was associated with decreased odds of Pap smear testing compared with women with a normal BMI (ORs for Pap testing: 0.91 for overweight, 0.81 for class I obesity, 0.75 for class II obesity, and 0.62 for class III obesity) [8].

In addition, even when obese women have the opportunity to be screened, difficulties in getting an adequate or good-quality biopsy from these women owing to a lack of appropriately sized examination equipment may result in unreliable Pap test results [41]. Second, the vast majority of cervical cancers are squamous cell cancers; however, 10%–20% are adenocarcinomas, and the incidence of adenocarcinoma has been increasing, both in absolute number and in proportion to squamous carcinomas [42]. Cervical adenocarcinoma may represent a more hormonally responsive cancer, and there could potentially be a mitogenic effect of increased estrogen on glandular cervical cancers [7]. Adenocarcinoma may be associated more strongly with obesity and body fat distribution than squamous cell carcinoma [43], [44]. However, our study did not provide information on the histologic subtype; additional studies, including analysis of the association between obesity and specific histologic types of cervical cancer, are needed.

In general, Asian people (but not the Chinese) have a lower BMI for the same age and sex than the Western white population [45]. Nevertheless, the proportion of Asians with a high risk of type 2 diabetes and cardiovascular disease is substantial, even in those with BMI below the existing WHO cutoff point of 25 kg/m^2^ [46], [47]. Data from nonsmoking Koreans showed that those with a relatively higher (24.0–25.9 and 26.0–27.9 kg/m^2^) BMI had an increased risk of stroke (adjusted HR [95% CI], 1.3 [1.0–1.5] and 1.4 [1.2–1.8], respectively) than those with BMI within the reference range (22.0–23.9 kg/m^2^), after adjustment for the possible effects of obesity [48]. Other data in mainland Chinese [49] and Indians [50] showed that the relative risk of having at least 1 risk factor for cardiovascular disease is high at a low BMI. The range for acceptable, normal, or optimum BMI for Asian populations should be narrowed to 18.5–23 kg/m^2^, according to a WHO expert consultation on appropriate BMI for these populations [51]. The current WHO cut-off points of 18.5, 25, 30 and 40 kg/m^2^ are retained. But the cutoff points of 23, 27.5, 32.5 and 37.5 kg/m^2^ are to be added as points for public health action [52]. There have been two previous reports to interpret the WHO BMI cut-offs in Asian and in Pacific populations [53], [54]. A proposal has been made to redefine the classification of obesity using BMI for Asian population as there is evidence to support that the increased risks of co-morbidities with obesity occurs at a lower BMIs in Asians [53]. Based on the recommendation of WHO Expert Consultation (2004) and Korean Society for the Study of Obesity, cut-points for overweight and obesity used in in this study was as follow: less than 18.5 kg/m^2^, underweight; 18.5–23 kg/m^2^, normal; 23–25.0 kg/m^2^, overweight; and 25.0 kg/m^2^or higher, obesity. On the Korean National Health and Nutrition Examination Survey percent of the people with BMI ≥30 kg/m^2^ was rare as 3.4% (male 2.8%, female 3.8%). Actually, in our study, number of subject with BMI ≥30 kg/m^2^ is just 18 (1.6%): 8 in normal, 3 in CIN1, 4 in CIN3, and 3 in cancer. However people with BMI ≥25 kg/m^2^ are considered mild obesity on the basis of the current BMI cuff off points (obesity ≥30 kg/m^2^).

To our knowledge, no studies have reported on whether physical activity is associated with the development of CIN and cervical cancer. In this study, physical activity was inversely associated with CIN2/3 and cervical cancer, whereas it was not associated with CIN1; this finding has not been reported previously. Various mechanisms have been associated with several cancers, such as alterations in sex hormones and insulin growth factors, immune modulation, alterations in free radical generation, factors affecting body fat distribution, and direct effects on cancer [15], [18], [55]–[57]. Furthermore, a mechanism has not been clearly proposed for the role of physical activity in preventing cervical cancer and high-grade CIN. Exercise may act as an immune modulator that induces changes in the activity of macrophages, natural killer cells, lymphokine-activated killer cells, neutrophils, and regulating cytokines [57]. In our previous study, we reported that intralesional cytokines may be prognostic markers for clearance of high-risk HPV after 12 months of follow-up [58]. A persistent infection of high-risk HPVs may result in the development of high-grade cervical lesions [59].

Few studies have examined whether height, weight, and calorie intake are associated with the development of CIN and cervical cancer. Yoo et al. [26] reported that the risk of uterine cervical cancer was significantly increased in women of shorter height. However, in another study, a nonsignificant inverse association was found for height [25]. In our study, height was not associated with CIN and cervical cancer. The inconsistent results among studies may be due to the adjustment of different confounders. On the basis of the univariate analysis in this study, there was an inverse association between height and cervical cancer risk, which is consistent with the results from a previous study [26]. However, after adjustment for various confounders, especially BMI and physical activity, which were considered to be significantly associated with cervical cancer, height was not associated with cervical cancer. Some studies have reported that low body weight and calorie intake were associated with an increased risk for CIN and cervical cancer; however, the results were not statistically significant [25], [27]. In addition, the findings of our study showed that weight and calorie intake were not significantly associated with CIN and cervical cancer.

Our study has some potential limitations. The retrospective assessment of diet and physical activity may have an inherent measurement error. Although the average serving sizes were estimated, we did not ask about specific portion sizes. This method does not take into account the possible individual variations in portion size. Moreover, the results of the analysis of diet and physical activity might be inconsistent because the time frame for the FFQ was different from that of the physical activity questions. Another limitation is a possible recall bias. Reporting on the basis of histories may be biased because of the patients having knowledge of their diseases. Our study did not provide information on the specific histologic types of cervical cancer. Further research is needed to determine the possible effects of significant cervical cancer risk factors on the development of specific histologic types of cervical cancer. Finally, the lack of detailed information on several confounders such as socioeconomic status, sexual behavior, and infectious diseases is also a potential limitation of the study. Despite these limitations, the findings from our cohort study provide new information on the influence of physical activity on the development of CIN and cervical cancer. Physical activity was inversely correlated with high-grade cervical lesions and cervical cancer, and BMI was positively associated with cervical cancer risk. Our findings are consistent with a previous large cohort study, which reported metabolic risk factors and cervical cancer, including a significant positive association between BMI and risk of cervical cancer [60]. Therefore, in addition to screening for and treatment of CIN, recommendations on the maintenance of an appropriate BMI with an emphasis on physical activity may be an important preventive strategy against the development of cervical cancer.
